# Mitochondrial genome sequence analysis: A custom bioinformatics pipeline substantially improves Affymetrix MitoChip v2.0 call rate and accuracy

**DOI:** 10.1186/1471-2105-12-402

**Published:** 2011-10-19

**Authors:** Hongbo M Xie, Juan C Perin, Theodore G Schurr, Matthew C Dulik, Sergey I Zhadanov, Joseph A Baur, Michael P King, Emily Place, Colleen Clarke, Michael Grauer, Jonathan Schug, Avni Santani, Anthony Albano, Cecilia Kim, Vincent Procaccio, Hakon Hakonarson, Xiaowu Gai, Marni J Falk

**Affiliations:** 1 Center for Biomedical Informatics, The Children's Hospital of Philadelphia, Philadelphia, PA 19104, USA; 2 Department of Anthropology, University of Pennsylvania School of Arts and Sciences, Philadelphia, PA 19104, USA; 3 Department of Physiology, University of Pennsylvania Perelman School of Medicine, Philadelphia, PA 19104, USA; 4 Department of Biochemistry and Molecular Biology, Thomas Jefferson University, Philadelphia, PA 19107, USA; 5 Division of Human Genetics, Department of Pediatrics, The Children's Hospital of Philadelphia, Philadelphia, PA 19104, USA; 6 Computational Biology and Informatics Lab, University of Pennsylvania Perelman School of Medicine, Philadelphia, PA 19104, USA; 7 Molecular Genetics Laboratory, Department of Pathology, The Children's Hospital of Philadelphia, Philadelphia, PA 19104, USA; 8 Center for Applied Genomics, Department of Pediatrics, The Children's Hospital of Philadelphia, Philadelphia, PA 19104, USA; 9 Department of Biochemistry and Genetics, Angers University Hospital, School of Medicine, Angers, F-49000, France; 10 Division of Pulmonary Medicine, Department of Pediatrics, The Children's Hospital of Philadelphia, Philadelphia, PA 19104, USA; 11 Department of Molecular Pharmacology and Therapeutics, Loyola University Chicago Stritch School of Medicine, Maywood, IL, 60153, USA; 12 Department of Pediatrics, University of Pennsylvania Perelman School of Medicine, Philadelphia, PA 19104, USA

## Abstract

**Background:**

Mitochondrial genome sequence analysis is critical to the diagnostic evaluation of mitochondrial disease. Existing methodologies differ widely in throughput, complexity, cost efficiency, and sensitivity of heteroplasmy detection. Affymetrix MitoChip v2.0, which uses a sequencing-by-genotyping technology, allows potentially accurate and high-throughput sequencing of the entire human mitochondrial genome to be completed in a cost-effective fashion. However, the relatively low call rate achieved using existing software tools has limited the wide adoption of this platform for either clinical or research applications. Here, we report the design and development of a custom bioinformatics software pipeline that achieves a much improved call rate and accuracy for the Affymetrix MitoChip v2.0 platform. We used this custom pipeline to analyze MitoChip v2.0 data from 24 DNA samples representing a broad range of tissue types (18 whole blood, 3 skeletal muscle, 3 cell lines), mutations (a 5.8 kilobase pair deletion and 6 known heteroplasmic mutations), and haplogroup origins. All results were compared to those obtained by at least one other mitochondrial DNA sequence analysis method, including Sanger sequencing, denaturing HPLC-based heteroduplex analysis, and/or the Illumina Genome Analyzer II next generation sequencing platform.

**Results:**

An average call rate of 99.75% was achieved across all samples with our custom pipeline. Comparison of calls for 15 samples characterized previously by Sanger sequencing revealed a total of 29 discordant calls, which translates to an estimated 0.012% for the base call error rate. We successfully identified 4 known heteroplasmic mutations and 24 other potential heteroplasmic mutations across 20 samples that passed quality control.

**Conclusions:**

Affymetrix MitoChip v2.0 analysis using our optimized MitoChip Filtering Protocol (MFP) bioinformatics pipeline now offers the high sensitivity and accuracy needed for reliable, high-throughput and cost-efficient whole mitochondrial genome sequencing. This approach provides a viable alternative of potential utility for both clinical diagnostic and research applications to traditional Sanger and other emerging sequencing technologies for whole mitochondrial genome analysis.

## Background

MitoChip is an array-based platform that was first developed by Maitra et al (2004), in collaboration with Affymetrix, to sequence the entire human mitochondrial DNA (mtDNA) coding region (15,451 base pairs (bp)) [1]. Oligonucleotide probes were synthesized *in situ *and tiled over each coding position of both strands to interrogate its base composition. The subsequent version, MitoChip v.2.0, has expanded coverage across the entire human mitochondrial genome by adding redundant probes that target the most common haplotypes in the highly variable control region [2]. In their original paper, Zhou et al (2006) showed a very high reproducibility (>99.99%) of base calls for MitoChip v.2.0, with an average call rate of 94.6% (ranging from 89.2 to 96.8%). While the reproducibility and accuracy were impressive, the call rate they achieved was suboptimal for clinical diagnostic purposes. This finding alone has limited the wide adoption of MitoChip v.2.0, such that traditional Sanger sequencing and emerging Next Generation Sequencing technologies are typically preferred in a clinical diagnostic setting, despite their inherently higher cost and slower turn-around time.

Affymetrix GSEQ 4.1, which uses the Zhou et al. (2006) algorithm that was first implemented in RA tools [3], is the most commonly used software tool for analyzing MitoChip v2.0 data. Base calling with default settings in GSEQ 4.1 can achieve a call rate and accuracy in excess of 90% and 99.9%, respectively http://www.affymetrix.com. Several studies have shown that both metrics can be improved by varying several parameters, and that additional information can be obtained from redundant probes on the array. Most notably, an average call rate of 99.48% and accuracy of 99.98% were achieved in a comparative study [4] that used a set of bioinformatics filters developed by Pandya et al. [5].

Other efforts to develop an improved bioinformatics analysis pipeline for MitoChip v2.0 have met with only limited success. For example, in the method developed by Hartmann et al. [4], the call rate was improved by using the probe intensity values of redundant probes. A base call was determined when the ratio of the highest probe set signal intensity to background noise exceeded an empirically determined cutoff value. Unfortunately, this approach may improve the call rate without necessarily improving accuracy, and is more subject to batch effect when the background noise levels differ between sample sets.

Several additional challenges have limited the clinical utility of mtDNA sequence analysis using the MitoChip v2.0 platform. The first challenge is the detection of insertions or deletions (indels), particularly in regions known to have such variants. At present, the GSEQ 4.1 algorithm provides no explicit way to detect indels. A second challenge is an apparent lack of sensitivity and specificity to detect and report heteroplasmy. Since pathogenic mtDNA mutations may only be present in certain tissues and/or at certain percentage loads [6], the accurate detection and assessment of heteroplasmy levels are imperative to understand disease diagnosis and prognosis [7]. While the Affymetrix MitoChip v2.0 platform has the potential to detect heteroplasmy with great sensitivity [1], GSEQ 4.1 does not provide an efficient method for making accurate heteroplasmy calls [4]. A third challenge is the lack of a robust quality metric, as no measurement accurately captures the overall quality of each MitoChip data set. Call rate offers one reflection of robustness but can be misleading if used as the sole parameter of sample quality, since it is subject to influence not only by technical factors but also by biological factors such as the presence of indels. A final challenge is the absence of a base call summary method at regions of common variation that are captured by multiple probes placed on MitoChip v2.0.

Here, we describe the development and implementation of a custom bioinformatics pipeline that addresses these challenges. We assessed and validated the effectiveness of this pipeline using a carefully selected set of mtDNA samples. Our pipeline improves MitoChip v2.0 call rate without sacrificing accuracy and, at the same time, provides an effective means for assessing data quality, detecting large indels, and systematically identifying heteroplasmic mtDNA variants.

## Implementation

**Figure 1:**
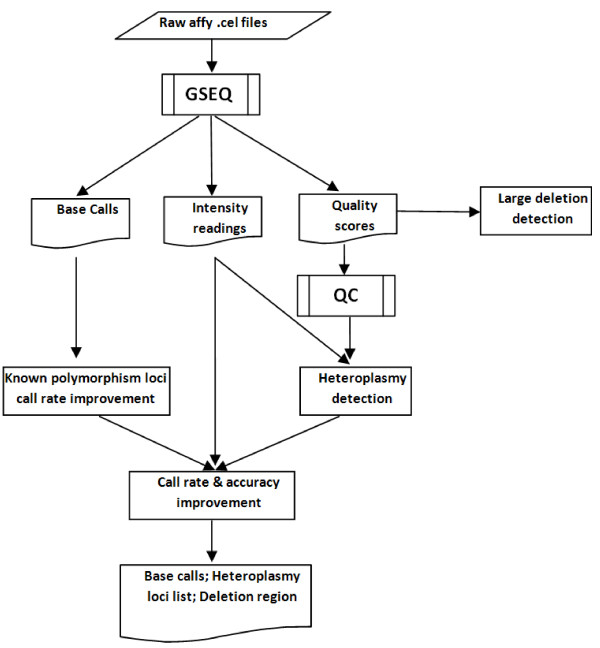
**MitoChip filtering protocol (MFP) workflow diagram**.

We designed and developed a customized bioinformatics pipeline, MitoChip Filtering Protocol (MFP), to analyze mtDNA sequence data generated by Affymetrix MitoChip v2.0 (Figure 1). The methods and algorithms used in the study were implemented as MATLAB code that is publicly available at MATLAB Central file exchange using the keyword "MFP" http://www.mathworks.com/matlabcentral/fileexchange/ (Additional File 1).

### DNA Sample Description

A total of 24 human mtDNA samples were studied (Table 1). These DNA samples were extracted by standard techniques from whole blood (18 samples), skeletal muscle (3 samples), or cell lines (3 samples). Two of the DNA samples were extracted from different tissues of the same individual that had variable heteroplasmy loads (samples #7 and #8 from muscle and whole blood, respectively). Eighteen of the samples were previously sequenced using conventional Sanger sequencing on a clinical basis at either the Baylor Mitochondrial Laboratory (8 samples by whole mtDNA genome sequence analysis) or on a research basis by the authors (10 samples), following the methods of Palanichamy et al. [8]. Two other samples had previously been studied only with a common mtDNA point mutation panel at Baylor Mitochondrial Laboratory (samples #1 and #2). Three additional samples were previously analyzed by denaturing HPLC-based heteroduplex analysis on a clinical basis at Transgenomics Laboratory (samples #3, #4, and #5). The last sample (#15) was previously processed using Illumina Genome Analyzer II at the University of Pennsylvania. Known pathogenic mutations in this data set included a five kilobase (kb) pair deletion (#14), and six known heteroplasmic mutations (#2, #6, #8, #17, #18, and #21). Samples #16 through #24 were selected to represent a wide range of common and rare mtDNA haplogroups (V7, H11, U4a1, D5a, J1c2, T1a, U4b3, and D5c). A total of 5 muscle DNA samples previously analyzed by Sanger sequencing were used as an independent, validation data set.

**Table 1:** Sample Characteristics.

Sample ID	Group	TISSUE ORIGIN	Whole mtDNA SEQUENCING PREVIOUSLY PERFORMED	COMPARATIVE SEQUENCING METHOD	mtDNA Haplogroup	MFP Predicted Haplogroup	Unique or Pathogenic Known Feature	Heteroplasmic variant levels	MitoChip Detection of a priori known feature(s)	MFP MitoChip v2.0 Call Rate (%)
1	Clinical	Blood	No	Common point mutation panel (Baylor)	H	H	None		Yes	99.5
2	Clinical	Blood	No	Common point mutation panel (Baylor)	I2	I	Heteroplasmic 3243A > G	84%	Yes	99.7
3	Clinical	Blood	No	DHPLC (Transgenomics)	B2b	R*	None		Yes	99.6
4	Clinical	Blood	No	DHPLC (Transgenomics)	R*	R*	None		Yes	99.5
5	Clinical	Muscle	No	DHPLC (Transgenomics)	N1b2	N1*	None		Yes	99.7
6	Clinical	Muscle	Yes	Sanger (Baylor)	J1c	J	Homoplasmic 10845C > T Heteroplasmic 5049C > T	Not reported	Yes	99.8
7	Clinical	Muscle^&^	Yes	Sanger (Baylor)	J1c	J	Homoplasmic 12264C > T	100%	Yes	99.6
8	Clinical	Blood^&^	No	qPCR of heteroplasmic variant (Baylor)	J1c	J	Heteroplasmic 12264C > T	30%	Yes	99.6
9	Clinical	Blood	Yes	Sanger (Baylor)	N1a	N1*	Homoplasmic T insertion between 5537 and 5538		Yes	99.8
10	Clinical	Blood	Yes	Sanger (Baylor)	N1b2	N1*	None		Yes	99.7
11	Clinical	Blood	Yes	Sanger (Baylor)	W1c	W	Homoplasmic 11204T > C		Yes	99.6
12	Clinical	Blood	Yes	Sanger (Baylor)	L1b1a	L0/L1	Homoplasmic 11778G > A		Yes	99.5
13	Clinical	Fibroblast Cell Line	Yes	Sanger (Baylor)	H	H	None		Yes	99.6
14	Research	Cell line	Yes	Sanger (MPK)	K	L3	5 Kb deletion		Yes	N/A
15	Research	Hela Cell Line	Yes	Illumina GAII (JAB)	L3b1a1	L3	None		No	99.7
16	Research	Blood	Yes	Sanger (TGS)	V7	V	None		Yes	99.7
17	Research	Blood	Yes	Sanger (TGS)	H11	H	Heteroplasmy 9966G > A	20%	No	98.5
18	Research	Blood	Yes	Sanger (TGS)	U4a	U*	Heteroplasmy 1706A > G	25%	No	98.9
19	Research	Blood	Yes	Sanger (TGS)	D5a	L3	None		Yes	99.7
20	Research	Blood	Yes	Sanger (TGS)	D5a	L3	None		Yes	99.6
21	Research	Blood	Yes	Sanger (TGS)	J1c2	J	Heteroplasmy 12879C > T	45%	Yes	99.7
22	Research	Blood	Yes	Sanger (TGS)	T1a	T	None		Yes	99.6
23	Research	Blood	Yes	Sanger (TGS)	U4b3	U*	None		Yes	99.8
24	Research	Blood	Yes	Sanger (TGS)	D5c	L3	None		Yes	99.6

13 clinical samples and 11 research samples were analyzed by MitoChip v2.0. Tissue origin, comparative mtDNA genome sequencing methodologies, unique or pathogenic features that characterize particular samples based on a priori sequencing knowledge, variant heteroplasmy levels, as well as MitoChip performance in terms of ability to detect known variants and the call rate achieved using MitoChip Filtering Protocol (MFP) are detailed. The 'mtDNA haplogroup' column details the manually curated haplogroup based on full sequence analysis for each sample. 'MitoSNP predicted haplogroup' was based on a subset of 22 mtDNA positions and generally agreed with manual curation, with the exception of haplogroups B, K, and D that were not properly identified by MitoSNP prediction. ^&^, muscle and blood samples originated from the same subject.

### MitoChip v2.0 Sequencing

All 24 DNA samples detailed in Table 1 were processed in the Center for Applied Genomics at The Children's Hospital of Philadelphia using the Affymetrix GeneChip Human mitochondrial resequencing array version 2.0 (MitoChip v2.0) per standard Affymetrix protocol [2].

### Illumina Genome Analyzer II Sequencing

The mtDNA library for Sample #15 was sequenced to 36 bp (single end) using an Illumina Genome Analyzer II. The resulting 9,760,673 reads were aligned using ELAND (pipeline version 1.30) to the human genome reference sequence (hg18) including the mitochondrial genome allowing up to 2 mismatches. All reads that did not align or aligned to mtDNA (chrM) and had fewer than 5 alignments at 0, 1, and 2 mismatches were selected. This step yielded 6,673,682 mtDNA candidate reads. These reads were assembled using Velvet (0.7.31 PMID 18349386) with an overlap setting of 31. Velvet produced 57 contigs, which were aligned to the hg18 'chrM' using fasta35 to assess coverage. A consensus sequence was also generated with MAQ (easyrun mode v0.7.1 http://maq.sourceforge.net/) using the same set of input sequences and the hg18 chrM sequence as a reference.

### GSEQ 4.1 Analysis

RAW data (cel files) of the 24 MitoChip v2.0 data sets were initially processed with GSEQ 4.1 software using both diploid and haploid models. All default parameters were used with the exception of the quality score threshold, which was set at 3 rather than the default setting of 12 to optimize sensitivity and specificity [4]. Base calls were made for each sample and position, which included "A", "C", "G", and "T". "N" was used to indicate when no base call could be confidently made because the quality score was below 3. Aside from base calls, the sequences and intensities of each probe were exported and analyzed for additional base and heteroplasmy calls.

## Results

### Quality control (QC) analyses

**Figure 2:**
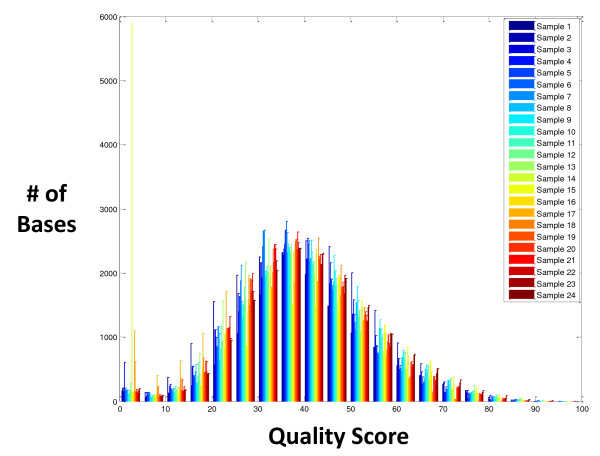
**Quality score distribution for all 24 samples**. Quality score distribution is shown for all bases of all 24 samples. Sample #14 has a uniquely low quality score distribution.

The call rate of a genotyping array is generally considered to be a good indicator of data quality but can potentially be misleading when influenced by technical factors or biological contributions such as pathogenic indels. In addition, using a threshold by which to identify poor quality samples can be subjective. Therefore, we devised a procedure that used call rate and base call quality correlation coefficient to identify potentially problematic samples. The generalized extreme studentized deviate (GSD) many-outlier method was then applied in the following manner to eliminate any outliers that had poor data quality [9]. First, we plotted the quality score distribution of all bases for each of the 24 DNA samples (Figure 2). As expected, quality scores were normally distributed for all samples except for the one that had a known 5-kb deletion (#14). While a large number of bases in this sample had the lowest quality score, the remaining bases in this sample appeared to have normally distributed quality scores. This observation correlated with the large mtDNA deletion known to be present in the sample.

**Figure 3:**
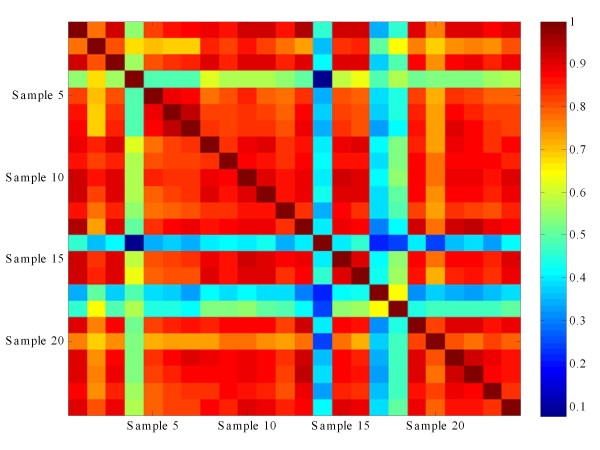
**Correlation heat map for all 24 samples**. The heat map plots the correlation coefficient score between any two samples. Samples #4, #14, #17, and #18 were clear outliers relative to the other samples.

Next, correlation coefficients of quality scores between different samples were analyzed (Figure 3). While it was expected that sample #14 would correlate poorly with other samples due to its large deletion, three additional samples (#4, #17, and #18) showed similar evidence of poor correlation. Thus, correlation coefficient analysis was strongly indicative of the overall poor quality of these four samples. Additionally, while their overall quality score distributions were similar to that of other samples, peaks at low-quality ends could clearly be seen for these samples, albeit to a much lesser degree compared to sample #14.

**Figure 4:**
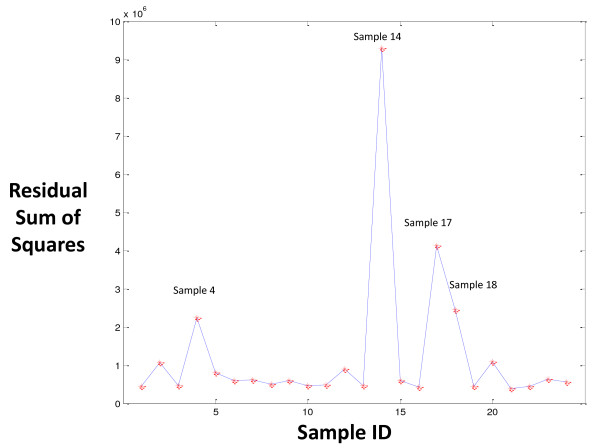
**Residual sum of squares (RSS) plot for all 24 samples**. Using the mean quality score across all samples for each probe as the baseline, the sum of squares of the difference between quality score for each sample and the baseline was determined. Samples #4, #14, #17, and #18 were outliers as had been shown by the generalized extreme studentized deviate (GSD) many-outlier procedure performed in Figure 2 [9].

Finally, the mean quality score across all samples for each probe was used as the baseline for purposes of computing the sum of square of the difference between each sample and the baseline (Figure 4). As before, samples #4, #14, #17, and #18 were found to be outliers from the remaining twenty samples. Call rates for those samples originally made using a quality score cutoff of 3 in GSEQ were all below 97% and represented a clear departure from the remaining samples that had call rates of approximately 99%. This observation supported the interpretation that these samples were problematic and should not be included in downstream analyses.

**Figure 5:**
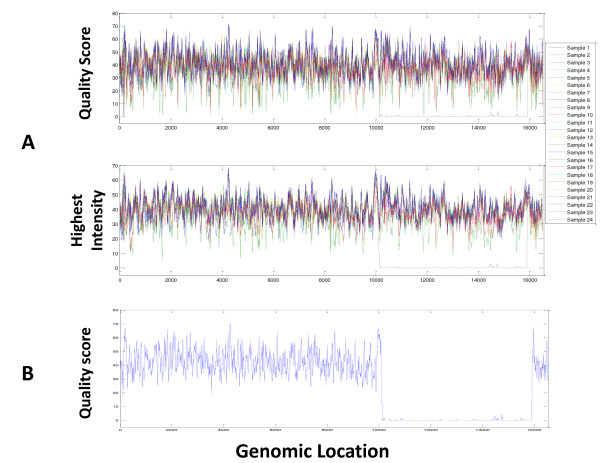
**Quality score analysis**. (A) (Top) Average quality score plots for all 24 samples, using a 25 bp moving window. (Bottom) Highest intensity value plots for all 24 samples, using a 50 bp moving window. (B) An average quality score plot is shown for a single sample (#14) using a 25 bp moving window.

The atypical nature of sample #14 became more evident when the average quality score was plotted across its mtDNA genome using a 25 bp moving window for all samples. In this analysis, samples with a deletion or insertion were expected to show a significant dip in the plot at the corresponding genomic region. Indeed, this is exactly what was seen for sample #14 (Figure 5). While the rest of that sample's mitochondrial genome behaved similarly to the other study samples in terms of the distribution of mean quality scores, consecutive stretches ranging from nucleotide positions (np) 10152 to 15945 had extremely low quality scores (Figure 5a, top panel). A continuous and significant drop of the highest probe intensity was also seen across the same region using a 25 bp moving window (Figure 5a, bottom panel). The pattern became more obvious when the quality score plot for this sample was individually studied (Figure 5). Outside of the deleted region, however, base calls were 100% concordant between MitoChip v2.0 and Sanger sequencing.

### Structural variant detection

The large 5791 bp deletion in sample #14 located between np 10154 and 15945 had previously been identified by Sanger sequencing. This result indicated that precise structural variants can be reliably detected and defined from MitoChip data simply by plotting either the average probe quality scores or the highest probe intensity values. Break-point determination with this approach was also highly accurate, as coordinates of the predicted deletion only differed by 2 bp on one side. This result supported the feasibility of detecting at least large size deletions using a moving window approach. At the same time, our failure to detect a 2 bp AC deletion in the control region of sample #15 demonstrated the limitation of this method for identifying structural variants at finer resolution. Unfortunately, a series of samples with indels of different sizes were not included in the study samples to permit experimental determination of this method's sensitivity.

However, we did explore a computational approach to predict the estimated size of structural variation that could potentially be detected by our moving window approach, (Additional File 2). We started by developing a statistically rigorous measure of a deletion, rather than using visual inspection alone (as illustrated in Figure 5), that involved grouping and collectively assessing the quality scores from all samples having no known deletions. A quality score that was 3 standard deviations below the mean was chosen as the cutoff. If the average quality score of a stretch of consecutive bases fell below this cutoff, this segment was deemed to be deleted. In our set of samples, the cutoff value was determined to be 14.86, which allowed us to determine that the deletion in sample #14 was located from np 10154 to 15943.

Because of the lack of samples with various sizes of deletions, we estimated the sensitivity of our method using simulation tests. Briefly, we first randomly chose a segment of a given size from the known deletion of sample #14 and used its data to replace those of a segment of the same size randomly chosen from another sample. We then applied our method to assess whether or not this segment was detected as a deletion. As seen in Additional File 2A, deletions that were 25 bp or longer could be clearly detected. Deletions that were smaller than 25 bp in length could still be detected but with increasing difficulty as their size decreased (Additional File 2B). To quantify the sensitivity of our method for deletions 2-24 bp in length, we repeated 200 simulations for each given size using the global cutoff value of 14.86 as described before. The fraction of times that we could detect the deletion was estimated as the sensitivity of our method. As shown in Additional File 2C, the sensitivity was as high as 90% for deletions of 18 bp or larger, but as low as 20% for deletions of 10 bp or shorter.

### Limitations to base call rate in GSEQ 4.1

GSEQ 4.1 sets the default quality score cutoff at 12. When keeping all other parameters at default values but lowering the quality score cutoff to 3, an average call rate of 99.0% was achieved for the 20 samples having consistently high quality scores. The call rate for those 20 samples ranged from 98.3% to 99.3% with a standard deviation of 0.22%, which is still not high enough to consistently satisfy the rigorous requirements of clinical diagnostic applications. While the call rate could be improved by further lowering the quality score setting, this would also increase erroneous base calls. Thus, it was evident that a method was needed to improve the call rate without sacrificing accuracy.

### Incorporating intensity values to improve call rate

GSEQ 4.1 software uses the quality score as the sole threshold by which to determine base calls. This standard leads to greater confidence being placed on base calls with higher quality scores. However, we found that quality score was highly correlated between samples, where certain loci had proportionally lower quality scores than did others. Therefore, relying solely on quality scores to determine base calls may diminish call rates for certain regions of the mitochondrial genome.

To further improve call rate, we incorporated into our pipeline an advanced analysis of intensity values that were determined in GSEQ 4.1 software. Rather than simply taking the probe with the highest intensity value at a given position, a call was made if the probe that had the highest intensity of all probes significantly exceeded background noise. While many approaches can estimate background noise, the second highest intensity value from both strands was used to provide conservative noise estimations. The ratio between the highest and second highest intensity values from both strands was used to measure confidence in generating additional base calls. A threshold was selected based on our statistical analysis of the ratio distribution.

Sample #14, which contained the large 5.8 kb deletion, provided a good measure of signal to noise ratio reliability based on thousands of data points, since the signals from deleted loci could all be considered as noise. The ratios of the deleted bases in this sample were closely analyzed (Additional File 3). The median plus four times the standard deviation of the inter-quartile value (1.26) was chosen as the threshold to differentiate outliers or true signals from pure noise. For all other samples, bases with non-calls ("N") were reexamined. If the signal-to-noise ratio exceeded 1.26, then a call was made according to the probe with the highest signal. Otherwise, the non-calls remained as they had initially been defined. In addition, any single nucleotide variant (SNV) calls having a quality score below 12 were converted to "N". Our revised analytical method was then applied to SNV calls across the entire mitochondrial sequence of each sample, where any probe having the highest to second highest signal intensity ratio less than 1.26 were converted into non-calls.

### Analysis of base calls in common variable regions

Affymetrix MitoChip is a sequencing-by-genotyping technology. It relies upon the signal intensities of a set of 25-mer probes that hybridize to the DNA sample and specifically interrogate the base composition at the position corresponding to the 13^th ^position of these probes. Its performance is complicated by the existence of many common mtDNA sequence variants, especially in the control region. Probes that do not match perfectly with the DNA sample at the bases beside the 13^th ^position could therefore potentially lead to ambiguous calls or non-calls. To address this problem, redundant probes were tiled over the common variable regions on the Affymetrix MitoChip v2.0. Details of the design can be obtained from the Affymetrix website http://www.affymetrix.com. Unfortunately, GSEQ 4.1 does not provide an effective and integrated way to use these data.

An algorithm called ReseqChip was recently developed to improve the call rate in these regions [10], but uses very complex methodologies to do so. We employed a similar but much simpler scheme. For a given position in a polymorphic region, all base calls for all probes having a quality score above 3 were collected and a majority vote was used as the final base call. In cases when two different base calls had an equal number of votes, a rank sum test of their quality score was examined. If the resulting p-value was less than 0.05, then the base call with the higher average quality score was chosen as the final base call. Bases with a p-value higher than 0.05 were further examined for potential heteroplasmy.

### Combined improvement in call rate achieved using MFP

**Figure 6:**
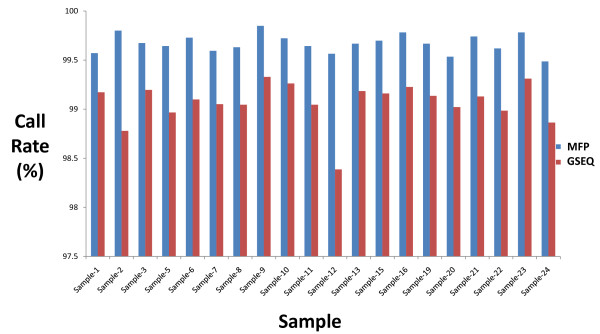
**Comparison of Call Rate by MFP and GSEQ 4.1**. Call rate comparison for each sample processed by MFP and GSEQ 4.1 alone.

**Figure 7:**
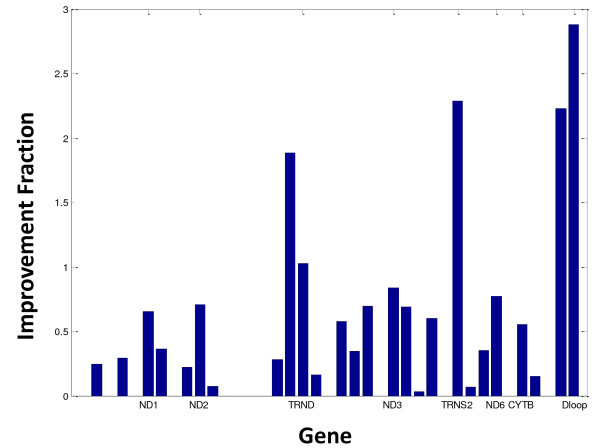
**Improvement fraction in call rate by gene**. Average call rate comparison by gene for each sample processed with MFP over GSEQ 4.1 alone.

All of the above-mentioned methods were integrated into a single MitoChip data analysis pipeline, which we called the MitoChip Filtering Protocol (MFP). Significant improvement in the call rate was obtained in MFP relative to that generated in GSEQ 4.1 (Figure 6). For the 20 samples that passed our QC filtering and did not have a large deletion, the average call rate achieved was 99.75% (range: 99.4-99.8%). By contrast, the average call rate for the same data sets using GSEQ 4.1 by itself was 99% (range: 98.3%-99.3%). Interestingly, different magnitudes of improvement in call rate were seen in different gene regions for different samples (Figure 7). As expected, significant improvement was seen in all samples in the control region attributable to the improved use in MFP of redundant control region probes.

### Accuracy of base calls generated using MFP

To assess the accuracy of base calls generated in MFP, we compared calls for 14 samples that had previously been analyzed by either Sanger or Illumina GAII next generation sequencing. A summary of the variant results by sample is provided in Table 2. On average, we observed two discordant base calls per sample, which translates to approximately 99.99% concordance rate between MFP-analyzed MitoChip v2.0 data and traditional Sanger (or Illumina GAII) whole mtDNA genome sequencing methods. This calculation was based on the ability of MitoChip v2.0 to interrogate only 16,544 of 16,569 bp of the mtDNA genome.

**Table 2:** Variant discrepancy for calls made on MitoChip v2.0 with MFP bioinformatic analysis compared to other methods of whole mitochondrial genome sequencing.

**SAMPLE ID #**	**Total Discordant Base Calls (Missed/Extra)**
6	5 (0/5)
7	2 (1/1)
9	1 (1/0)
10	0 (0/0)
11	0 (0/0)
12	1 (0/1)
13	0 (0/0)
15	0 (0/0)
16	0 (0/0)
19	3 (2/1)
20	10 (2/8)
21	0(0/0)
22	0 (0/0)
23	3 (1/2)
24	4 (1/3)

Discrepant calls between MitoChip v2.0 using our custom MFP bioinformatics algorithm are summarized for each of the 15 study samples for which full mtDNA genome sequencing had been previously performed (samples #14, #17 and #18 were excluded from this comparative analysis due to consistent findings of poor sample quality on multiple analyses). Total number of discrepant calls is detailed, with numbers in parentheses specifying calls missed by MFP-based MitoChip analysis that had been made by standard sequencing, as well as the extra calls made by MFP-based MitoChip analysis that had not been detected by standard sequencing. The specific discordant variants from each sample are catalogued in Additional File 4.

The discordant base calls were closely examined (Additional File 4). MitoChip v2.0 data had high quality scores (≥12) using GSEQ 4.1 analysis for eight of the discordant bases that occurred at one of three nucleotide positions in the HVS1: 16360 (#19, #20), 16362 (#7, #19, #20, #24), and 16356 (#18, #23). We are confident that the 16362 and 16356 polymorphisms are present (i.e., they are not errors created during Sanger sequencing) because they are phylogenetically diagnostic mutations for the samples' respective haplogroups. Thus, these polymorphisms were clearly missed by MitoChip. Extra calls by MitoChip that were not seen in Sanger sequences or the NGS sequence were also noted. Six of the 14 mutations occur near known polymorphisms. Most of these mutations also involved unusual transversions. One of these transversions was found in two samples that belonged to two different mtDNA haplogroups (U4 and H), further suggesting that an error occurred in the MitoChip read.

The percentage of no-calls at cytosine (c) positions was consistently high across all samples (Additional File 4), as was similarly reported in prior studies [1,9,11]. Rates of "no-calls" were not linked to the mutational aspect of any specific haplogroup. In other words, there was no bias in mutation ascertainment towards phylogenetically older or younger haplogroups relative to one another. In addition, the majority of the extra calls appeared as atypical transversions, with many being adjacent to or near real polymorphisms.

We noted a high percentage of discordant calls for two samples (#17 and #18) relative to other mtDNAs in the sample set (Additional File 4). This difference may reflect the fact that they were the first samples that were processed and analyzed using MitoChip 2.0 in our data set. In spite of this problem, nearly all of the polymorphisms identified in the samples through Sanger sequencing were also detected by MitoChip 2.0, with most of the observed discrepancies being extra calls made by MitoChip 2.0. These results indicate that DNA samples collected 1-2 decades ago, such as #17 and #18, are amenable to sequence analysis with the MitoChip 2.0 and can produce very high call rates, despite their being of possibly slightly lower quality than recently collected samples.

### Improved sensitivity for heteroplasmy detection using MFP

**Figure 8:**
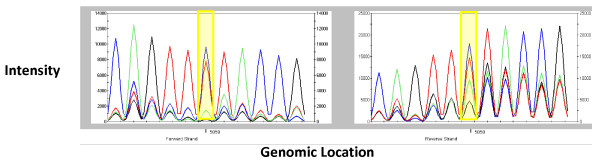
**Signal intensity pattern of heteroplasmic sites**. GSEQ intensity plots demonstrating identification of a known heteroplasmic site (yellow highlight) on both forward (left panel) and reverse (right panel) strands that had been originally demonstrated by Sanger sequencing. The peak at each position corresponds to the signal intensity of one of 4 probes (A,C,G,T) in a probe set.

A potential advantage of Affymetrix MitoChip array technology over Sanger sequencing technology is its higher sensitivity for heteroplasmy detection [1]. Since the heteroplasmy calls in GSEQ 4.1 often appeared to be questionable, as suggested by other studies [4], we developed our own statistical method for heteroplasmy detection. Intensity patterns of typical heteroplasmic sites are illustrated in Figure 8. The highest and second highest intensity values are close to each other but significantly differ from the other potential base calls. Importantly, we observed a high degree of correlation among probe intensities across different samples at the same position.

Therefore, we devised a mathematical approach to utilize the intensity values of multiple samples for purposes of detecting heteroplasmic sites. A ratio of intensity values at each position of a given sample was calculated as detailed in **Equation 1**:

(1) $$intensity_{new,i}=\log\left(\frac{intensity_{highest,i}}{intensity_{second\;highest,i}}∕\frac{intensity_{second\;highest,i}}{intensity_{lowest}}\right)$$

where *i *is the base position of interrogation. This equation measures the relative difference between the highest and second highest intensity value as compared to the second highest and lowest intensity value. At a heteroplasmic position, the second highest intensity would be much closer to the highest intensity but much more distant from the lowest intensity. Such ratios follow a bell-shaped distribution pattern, where extreme values to the left side of the distribution are suggestive of heteroplasmic loci.

To further eliminate false positive heteroplasmy detection, a four-step method was applied. First, since a true heteroplasmic site was assumed to have an extreme value both within a sample and across all samples, three standard deviations was used as a cutoff threshold. Second, any positions for which two different bases had the highest intensities on forward and reverse strands were filtered out. Third, potential false positives were filtered out using the base calls from GSEQ 4.1 that were made with either a diploid or haploid model. Calls were considered to be "real" only if the base calls were different using different models at a given base, which was another indication of a potential heteroplasmic site. Finally, we eliminated any heteroplasmic calls detected immediately adjacent to another detected SNP, since those heteroplasmic bases appeared to be false positives caused by neighboring positions to an imperfect match.

**Figure 9:**
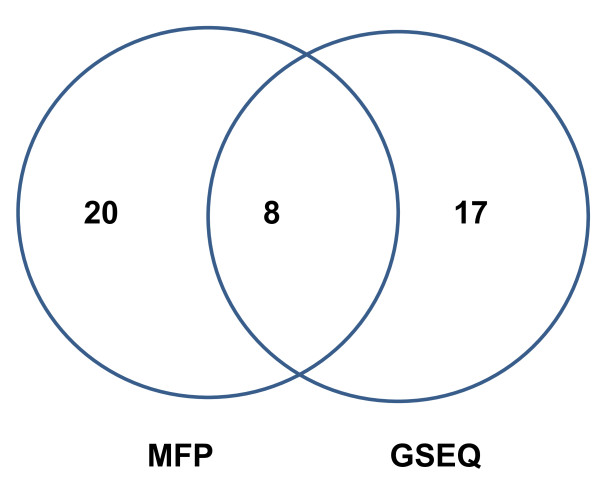
**Venn diagram comparison of heteroplasmy calls made by MFP and GSEQ 4.1**.

We applied this method to the 20 samples that passed quality control parameters to detect a total of 28 heteroplasmic sites in these samples, including four that had been previously identified by Sanger sequencing. We also assessed heteroplasmy calls with default GSEQ 4.1 settings for the same 20 samples. Of these variants, two of the six heteroplasmic loci identified by Sanger sequencing were missed by GSEQ 4.1. Sample #15 was sequenced using GAII with single-end 36 bp reads to provide an average 375X depth of coverage, which allowed for validation of some additional heteroplasmy calls that we had made in MFP. For this sample, GSEQ 4.1 detected one heteroplasmic mutation at np 3433. Examining the GAII sequence alignment using Tablet [12], we did not find any supporting evidence for this site being heteroplasmic, as it appeared to be entirely homozygous for thymine ("T") (Additional File 5). By contrast, our pipeline identified two potentially heteroplasmic loci from the same sample at np 15940 and 15944, which were supported by the GAII data (Additional File 6). Collectively, these results suggest that our MFP method for detecting heteroplasmy has a higher sensitivity and specificity than does GSEQ 4.1 alone (Figure 9). However, more extensive validation of the capability of MFP to detect heteroplasmy needs to be conducted, ideally using a next-generation sequencing technology for comparison.

### Heteroplasmy load quantitation using MFP

The heteroplasmy level at a given locus can be estimated using relative intensity ratios. For a detected heteroplasmic position, we first determined the relative ratio of two alleles and then calculated a product average of the two ratios, as per **Equation 2**:

(2) $${\bar{r}}_{i}=\sqrt{\frac{intensity_{allele1,i,sense}}{intensity_{allele2,i,sense}}\times \frac{intensity_{allele1,i,antisense}}{intensity_{allele2,i,antisense}}}$$

where ***i ***is heteroplasmy loci, and ***intensity_allele k, i, sense/antisense_***represents the intensity value of allele ***k ***at the sense or antisense strand. The ratio was then converted to the percentage of each allele type's abundance. Using this method, we estimated that the heteroplasmy levels for the A3243G mutation in sample #2 and for the C5049T mutation in sample #6 were 63% and 40%, respectively. Sanger sequencing followed by allele refractory mutation system (ARMS) based quantitative PCR [13] estimated the heteroplasmy mutation load for A3243G at 84% in sample #2. Heteroplasmy at the C5049T position was identified by Sanger sequencing but not further quantified in sample #6. Interestingly, MFP analysis identified heteroplasmy at np 5537 and 5538 for sample #9, which was reported to have an inserted thymine ("T") between these exact positions through Sanger sequencing. MFP-based MitoChip v2.0 analysis also detected similar heteroplasmy levels as estimated by Sanger sequencing alone or with ARMS qPCR, with these heteroplasmy loads being between 30% and 84%, respectively (Table 3). However, a heteroplasmic variant identified by Sanger sequencing in sample #18 at an estimated 25% heteroplasmy level was not identified by MFP analysis.

**Table 3:** Heteroplasmy detection levels made using the MFP analysis algorithm.

**Sample ID**	**mtDNA Genome Position**	**Heteroplasmy Level Sanger**	**Heteroplasmy Level MFP**
2	A3243G	84%	61%
6	C5049T	N.D.	34%
8	C12264T	30%	43%
9	5537	5537_5538 insT (100%)	58%
	5538		45%
17	G9966A	20%	Sample excluded
18	A1709G	25%	Sample excluded
21	C12879T	45%	55%

Sample #2, #6, #8, and #21 had heteroplasmic variants detected both by Sanger sequencing and MFP analysis of MitoChip v2.0 data. Of interest, the 5537_5538insT in tRNA-TRP identified by Sanger sequencing in sample #9 was interpreted in MFP as a heteroplasmic mutation at both positions 5537 and 5538. Although a heteroplasmic A1709G site was detected by Sanger sequencing in sample #18, this sample was excluded from MFP analysis due to poor sample quality. "N.D." = not determined.

### Validation data set analysis

We applied our custom MFP pipeline to an independent data set from five muscle DNA samples whose mtDNA genomes had been sequenced using both Sanger sequencing and MitoChip v2.0. Comparative results of MFP pipeline analysis and GSEQ 4.1 analysis results of MitoChip v2.0 data are detailed in Additional File 7. Overall, the MFP-based analysis of MitoChip v2.0 results closely matched those generated by Sanger sequencing, with an average of 1 base call error per sample being observed. It was noteworthy that the same base call discrepancy (A12307G) accounted for 3 of the 5 total SNP discrepancies identified across all five samples (Additional File 7C). The three samples affected all belong to mitochondrial haplogroup U, which has an A12308G change compared to the revised Cambridge Reference Sequence (Additional File 7D). These results highlight the limitations with MitoChip v2.0 design at a few individual loci, but further support the utility of the MFP pipeline to provide superior overall accuracy and call rate.

## Discussion

The Affymetrix MitoChip v2.0 resequencing array provides a relatively fast, high-throughput, cost-effective, and sensitive method for detecting both homoplasmic and heteroplasmic mutations across the entire human mitochondrial DNA genome. The costs for each MitoChip v2.0 array and reagents (currently estimated at less than $200/sample) are lower than any other commercially available mitochondrial DNA sequencing methodology. Furthermore, under a week is needed to sequence and analyze MitoChip v2.0 data using the MFP pipeline. By contrast, the current cost of sequencing the whole mitochondrial genome using Sanger sequencing or more recently available NGS platforms typically exceeds $2,000 per sample at a clinical diagnostic laboratory, and can take up to two months to obtain analyzed results. Furthermore, while GSEQ 4.1 is a standard software package designed to analyze MitoChip v2.0 data, it provides less than the desirable call rates necessary for clinical diagnostic applications in suspected mitochondrial disease.

In this study, we systematically analyzed the MitoChip v2.0 data from 24 carefully selected samples with a number of statistical methods. The outcome was the development of a custom pipeline, MFP, that significant improved the call rate and accuracy relative to GSEQ 4.1. With an average call rate of 99.75% across the entire genome and an estimated accuracy of 99.98%, MitoChip v2.0 analysis with the MFP bioinformatics pipeline can now be viewed as a viable and highly attractive alternative to Sanger sequencing.

A distinct advantage of Sanger sequencing over MitoChip v2.0 has been its ability to detect and precisely determine the breakpoints of indels of any size. In this study, we showed that, while indels of a few base pairs in size are still difficult to detect with MitoChip v2.0, deletions larger than 10 bp should be fairly straightforward to detect and precisely define. It is not clear whether the oligos in the Mitochip v2.0 can be further modified to detect smaller indels in mtDNA samples.

Since the MitoChip v2.0 platform was designed based on the revised Cambridge reference sequence that belong to mitochondrial haplogroup H [14,15], it has been suggested that MitoChip analysis would not reliably detect sequence variants from more divergent haplogroups, such as L0 [16]. As shown in Table 1, the 24 study samples analyzed here originate from a number of diverse haplogroups. If the data set passed QC control parameters, then a desirable call rate could be readily achieved in all cases. These data satisfactorily address concerns about the potential lack of sequence identification of the probes for divergent or rare mitochondrial DNA lineages.

While haplogroup origins could be precisely determined based on our manual curation of the MFP-determined mtDNA genome sequence, we note that implementation in MFP of an automated algorithm that relies solely upon a panel of 22 SNPs to assign haplogroup [17] failed to permit the accurate assignment of B (sample #3) or D5 haplogroups (samples #19, #20, #24). The haplogroup for sample #14 that had a 5.8 kb deletion was also understandably misassigned, as several of the haplogroup-defining variants for this sample (which belongs to haplogroup K) fell in the deleted region of this sample. Thus, haplogroup assignment can be readily made with MFP but must be viewed with caution if based on the 22 common SNP panel used here or if the sample harbors a large deletion. However, the use of an expanded set of SNPs representing a wide range of phylogenetically important markers from a global set of haplogroups will likely rectify these kinds of misassignments.

Another potential advantage of MitoChip v2.0 analysis over Sanger sequencing is its potential to more sensitively detect heteroplasmy. However, failure to exploit the data captured on MitoChip v2.0 relative to heteroplasmy detection and quantitation appears to be attributable to a limitation of the current GSEQ 4.1 and other software. This same problem has been noted in previous studies employing the MitoChip v2.0 [4]. While we were able to use MFP to detect 6 confirmed heteroplasmic bases (4 consistent with Sanger sequencing and 2 consistent with Illumina GAII), more extensive validation results are needed to determine the conditions that must be met to consistently achieve those calls across the mtDNA genome. MFP analysis did detect two potentially low-level heteroplasmic mutations in sample #15 at np 15940 and 15944, which were supported by deep sequencing data (Additional File 6). This result suggests that MFP can potentially make robust and accurate heteroplasmy calls.

Any array-based sequencing technology has inherent limitations that cannot be fully addressed by statistical or informatic means. Aside from the difficulty of detecting small indels, as mentioned above, another important issue is the reliable and consistent detection of very low levels of heteroplasmy. As newer technologies emerge, superior heteroplasmy detection will be achieved. In this regard, next-generation sequencing technologies offer enormous depth of coverage for the mitochondrial genome such that point mutations, small indels and low levels of heteroplasmy (at least 5%-10%) can be reliably and quantitatively detected [18-20].

Yet, current next-generation sequencing technologies are not without their own limitations. For example, such technologies are still relatively expensive in terms of equipment, reagents, and labor costs. Furthermore, they are high-throughput technologies only in terms of the amount of sequence data that they generate per run, but not in terms of the number of samples that can be individually processed at the same time. In contrast to NGS methods, MitoChip v2.0 analysis can be run on individual samples without having to accumulate a sufficient number of samples to batch analyze them.

## Conclusions

In summary, we conclude that MFP-based analysis of MitoChip v2.0 data provides a highly attractive option for both clinical diagnostic applications as well as research-based evaluation of the human mitochondrial DNA genome. The custom MFP bioinformatics pipeline reported here performs with consistently high call rate and accuracy, provides improved metrics to assess sample quality control, permits improved detection of the occurrence of both large deletions and heteroplasmic variants, and performs consistently well in samples of diverse haplogroup origins. With the bioinformatics algorithm improvements described here that we incorporated into a single MFP pipeline, MitoChip v2.0 analysis can now provide a fast, cost-effective, flexible, and truly high-throughput way of achieving comparable or higher accuracy relative to Sanger sequencing in the evaluation of a large number of individuals for potential pathogenic mutations throughout the mitochondrial genome.

## Availability and Requirements

Project name: MitoChip Filtering Protocol (MFP); Project home page: http://www.mathworks.com/matlabcentral/fileexchange/ using keyword "MFP"; Operating system: Platform independent; Programming language: Matlab; Other requirements: Matlab Statistics Toolbox; License: GNU GPL; Any restrictions to use by non-academics: None.

## Abbreviations

mtDNA: mitochondrial DNA; bp: base pair; kb: kilobase; SNV: single nucleotide variant; MFP: MitoChip Filtering Protocol; np: nucleotide position.

## Authors' contributions

MJF, XG, and TGS conceived of the project idea. MJF, EP, CC, and AS obtained clinical samples for analysis. JAB and JS provided the HeLA cell line sample and Illumina GAII sequence data from this line for comparison. MPK provided the 5.8 kb deleted cell line sample and comparative sequence file. TGS, MCD, MG, and SIZ selected and provided nine haplogroup samples for analysis, completed the Sanger sequencing of these samples for comparison, and made manual haplogroup calls for all samples. AA, CK, and HH performed all Affymetrix MitoChip v2.0 PCR and hybridization protocols of the original data set. VP provided the validation set sample data as analyzed by both Sanger sequencing and Affymetrix MitoChip v2.0. JCP and HMX established the custom bioinformatic pipeline and processed all MitoChip sample data. HMX, XG, TGS, MCD, and MJF wrote the manuscript. All authors read and approved the final manuscript.

## Supplementary Material

Additional file 1 **Source Code**. Click here for file

Additional file 2 **Structural variant detection capacity analysis in MFP**. **(A and B) **Quality score plots with 25 bp moving window for simulated data sets with deletion segments of different sizes (marked on the left). The deleted segment is highlighted in red in each plot. (**C) **Sensitivity plot for deletions of various sizes based on simulation tests. Click here for file

Additional file 3 **Box plot of ratios between the highest and second highest signal intensities of all bases located in the large deleted region of sample #14**. 12.7% of bases in the 5791 bp deleted region would fall above this cutoff. Click here for file

Additional file 4 **Single nucleotide variant discrepancies within 15 DNA samples analyzed both by Affymetrix MitoChip v2.0 with the MFP analysis algorithm and by either Sanger or Illumina Genome Analyzer II Sequencing methods.** Whole mtDNA genome sequence data was compared for all 15 high quality samples detailed in Table 2, as well as for 3 samples found to have poor quality (#14, #17, and #18). No sequence discrepancies were noted between MitoChip v2.0 and 7 of the high quality samples (#10, #11, #13, #15, #16, #21, #22), nor in sample #14 at any base positions outside of its large deleted region. Click here for file

Additional file 5 **Alignment of Illumina GA next generation sequencing reads from position 3433 in sample #15**. Mitochondrial genome position 3433, visualized in Tablet, shows no indication of heteroplasmy by next generation sequencing. Click here for file

Additional file 6 **Alignment of Illumina GA next generation sequencing reads from position 15940 to 15944 in sample #15**. The 2 base pair deletion located between np 15940 and 15944 in the mitochondrial genome, visualized in Tablet, clearly indicates these are potential heteroplasmic sites. Click here for file

Additional file 7 **Validation data set results comparison between MFP and Sanger sequencing**. **(A) **Call rate improvement when comparing MFP analysis to GSEQ 4.1 for each of 5 validation samples. **(B) **Accuracy between total calls made by MFP analysis and Sanger sequencing for each of 5 validation samples. **(C) **Call type details for a total of 5 discrepant calls between MFP analysis and Sanger sequencing seen in 3 of the validation samples. Interestingly, 3 of the 5 discrepant calls involved the same SNP (A12307G) in three distinct samples (C5, C9, C11) that was detected by MFP. **(D) **Sanger-based electropherogram clearly shows the presence of the A > G variation, not at 12307, but at its neighboring position 12308, as shown here for validation sample C5. Click here for file
