# Supplementary figures and images for: Mitochondrial genome sequence analysis: A custom bioinformatics pipeline substantially improves Affymetrix MitoChip v2.0 call rate and accuracy
Source: BMC Bioinformatics. 2011 Oct 19;12:402. doi: 10.1186/1471-2105-12-402 (PMC3234255; doi:10.1186/1471-2105-12-402)

## Slide 1
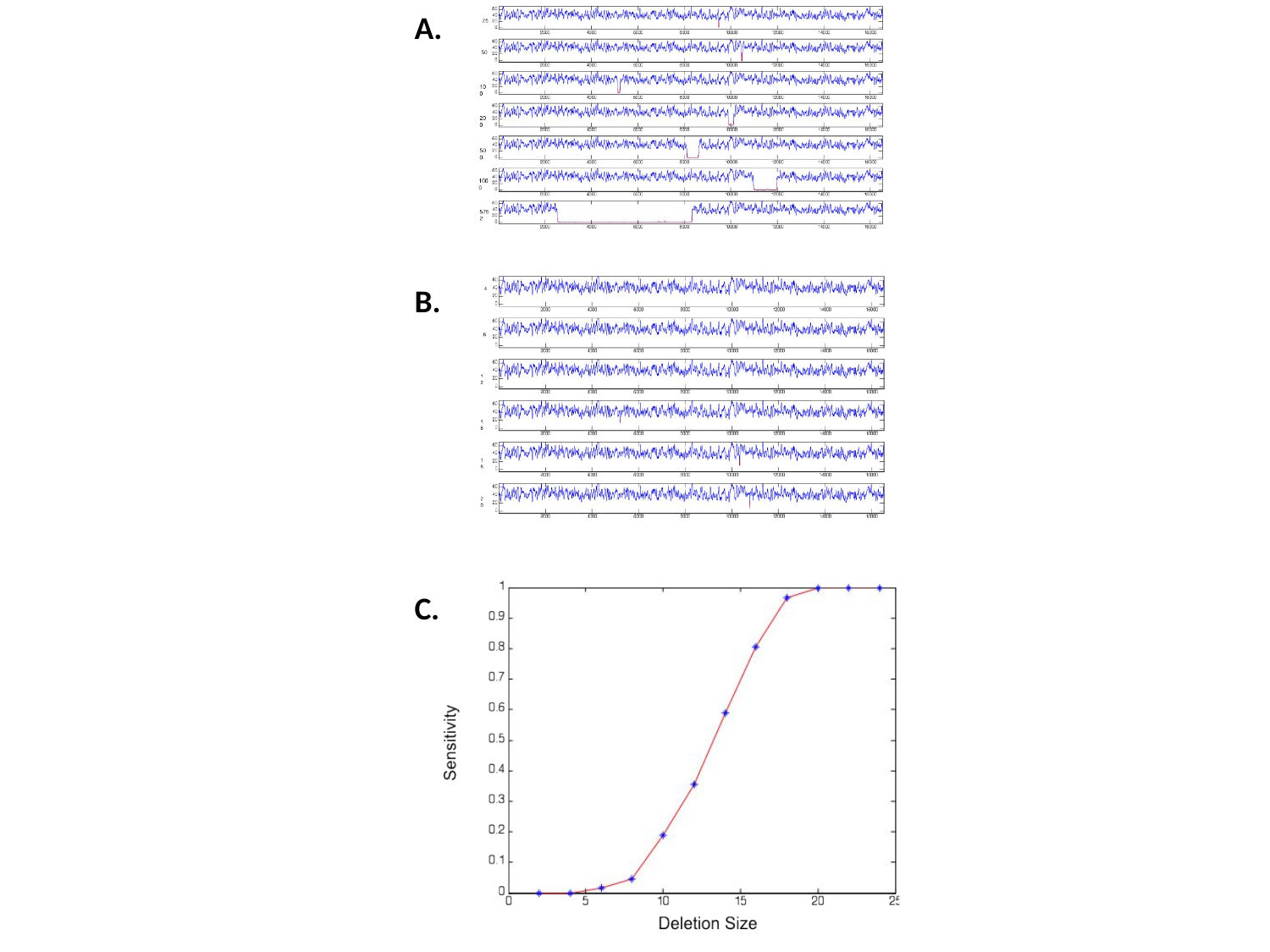

A.
B.
C.

Supplement: Additional file 2 — Structural variant detection capacity analysis in MFP. (A and B) Quality score plots with 25 bp moving window for simulated data sets with deletion segments of different sizes (marked on the left). The deleted segment is highlighted in red in each plot. (C) Sensitivity plot for deletions of various sizes based on simulation tests. [file 1471-2105-12-402-S2.PPT]

## Slide 1
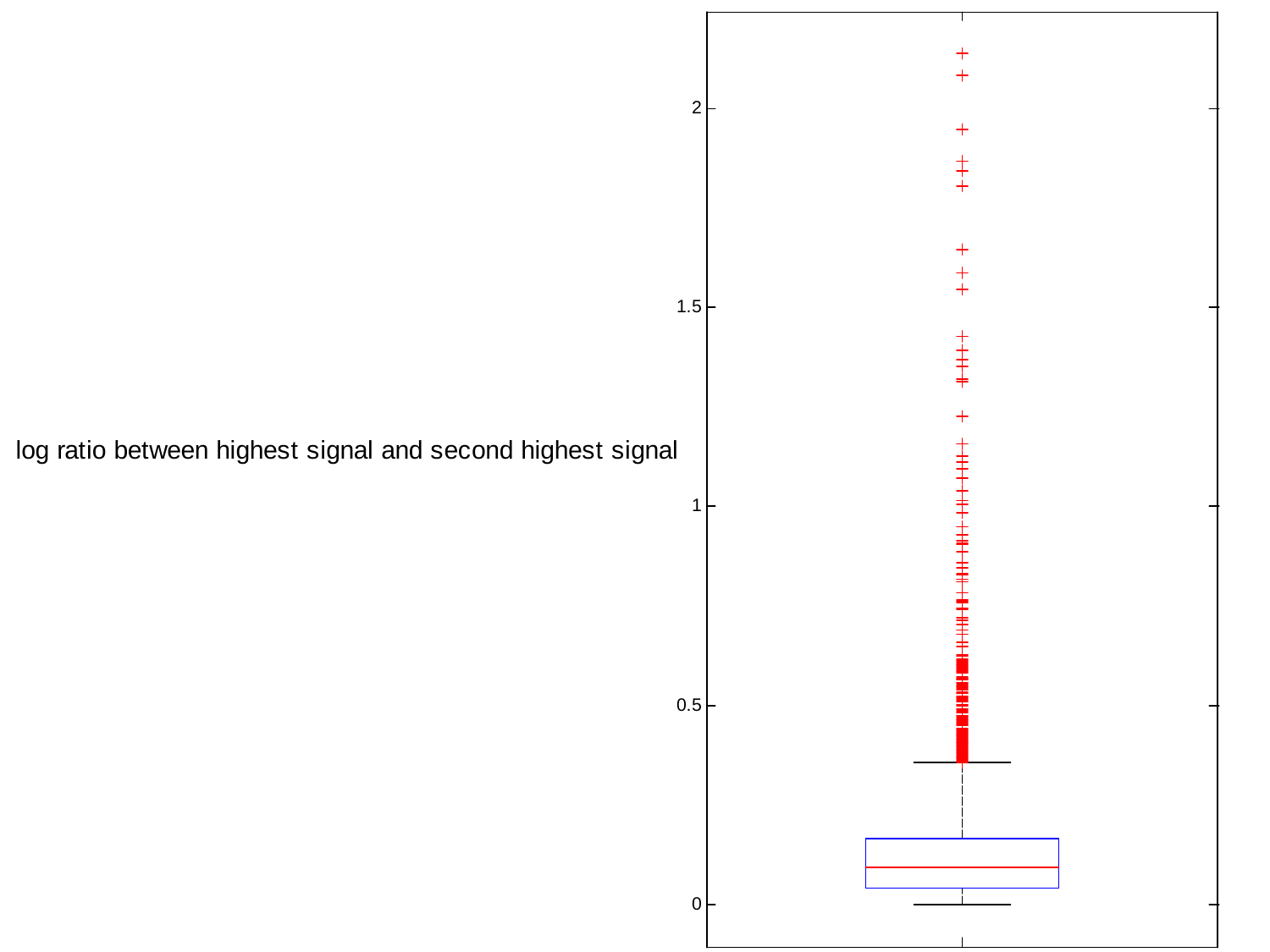

Supplement: Additional file 3 — Box plot of ratios between the highest and second highest signal intensities of all bases located in the large deleted region of sample #14. 12.7% of bases in the 5791 bp deleted region would fall above this cutoff. [file 1471-2105-12-402-S3.PPT]

## Slide 1
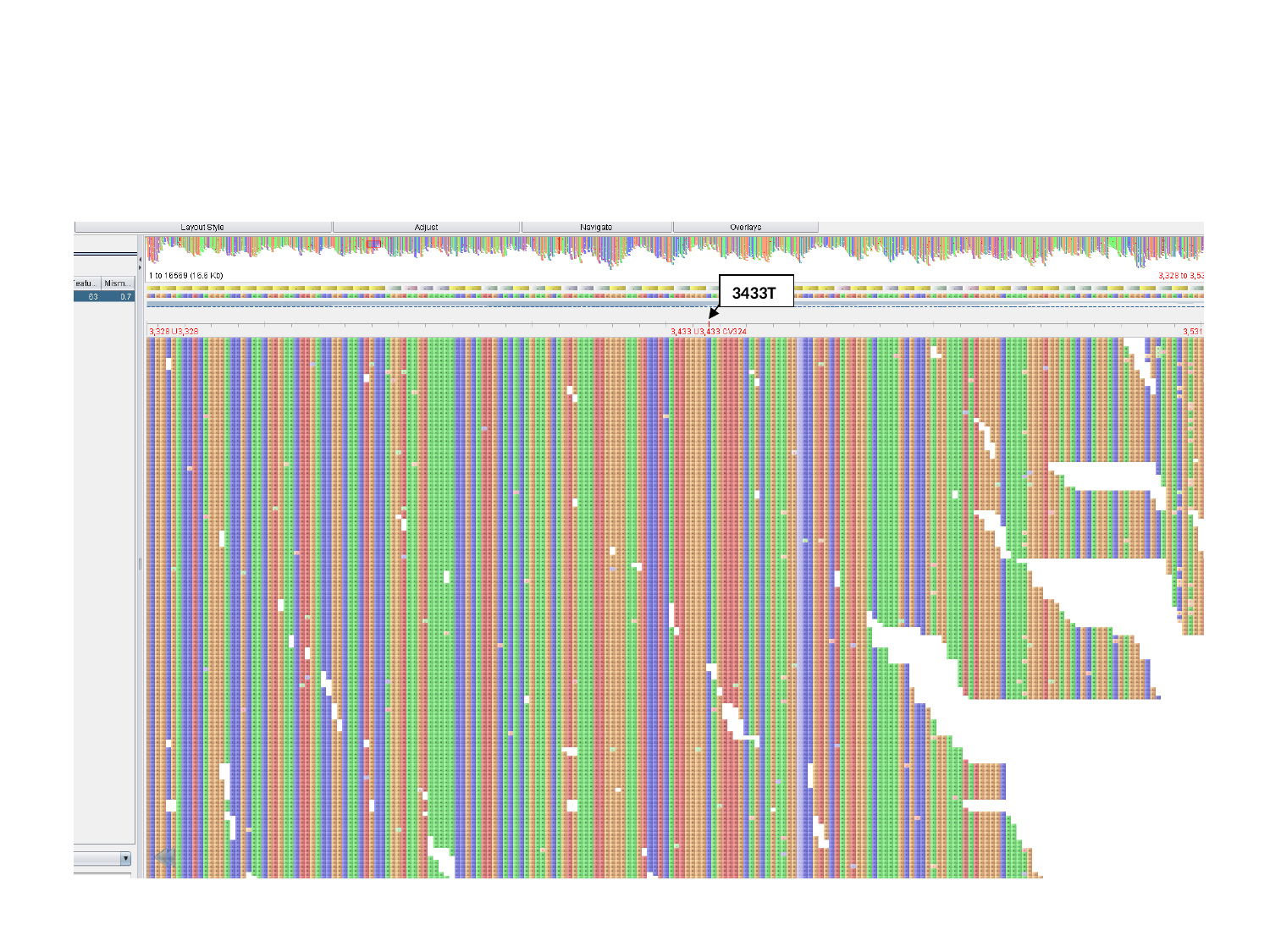

3433T

Supplement: Additional file 5 — Alignment of Illumina GA next generation sequencing reads from position 3433 in sample #15. Mitochondrial genome position 3433, visualized in Tablet, shows no indication of heteroplasmy by next generation sequencing. [file 1471-2105-12-402-S5.PPT]

## Slide 1
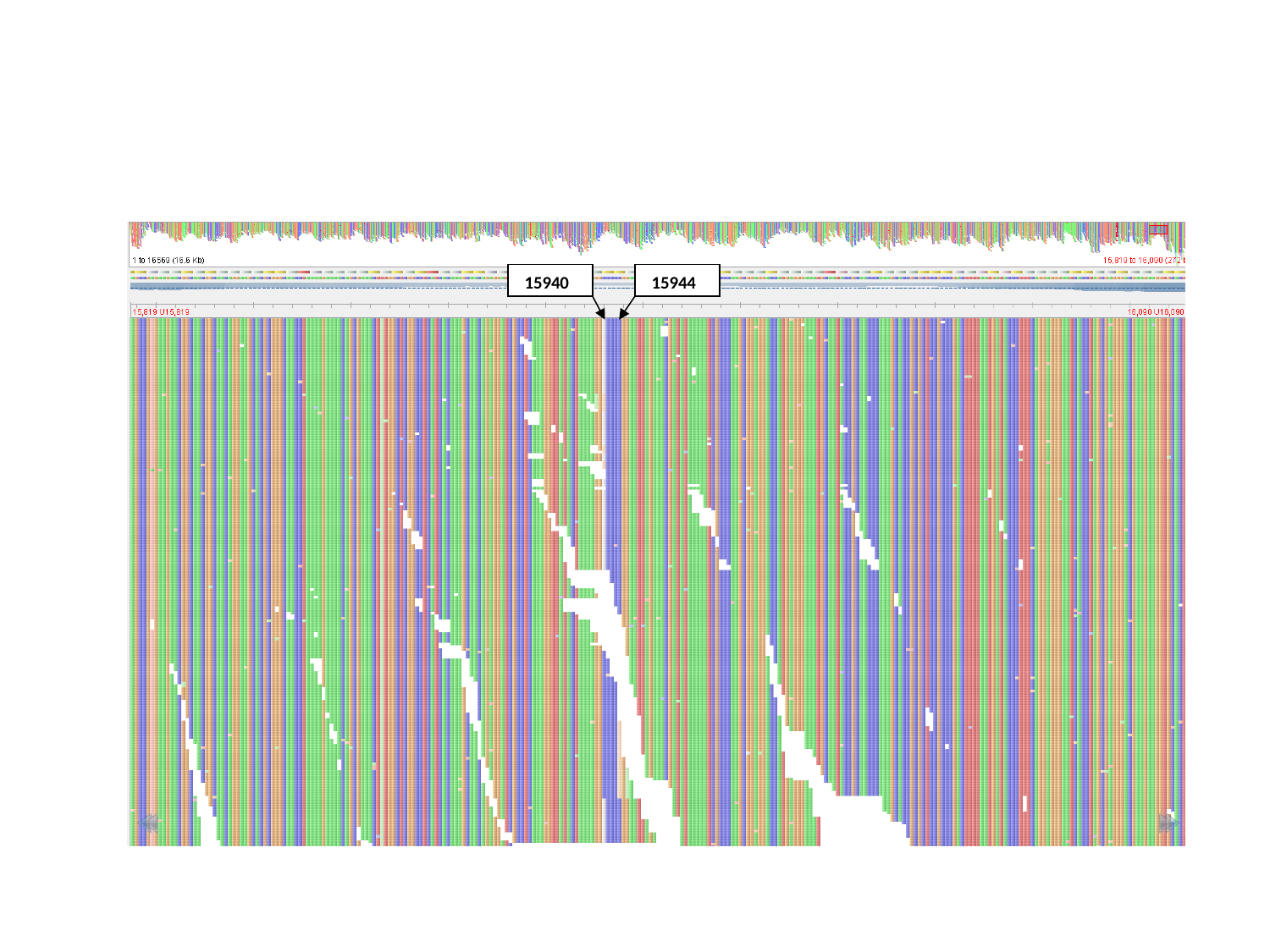

15940
 15944

Supplement: Additional file 6 — Alignment of Illumina GA next generation sequencing reads from position 15940 to 15944 in sample #15. The 2 base pair deletion located between np 15940 and 15944 in the mitochondrial genome, visualized in Tablet, clearly indicates these are potential heteroplasmic sites. [file 1471-2105-12-402-S6.PPT]
