# Supplementary material for: Mitochondrial genome sequence analysis: A custom bioinformatics pipeline substantially improves Affymetrix MitoChip v2.0 call rate and accuracy
Source: BMC Bioinformatics. 2011 Oct 19;12:402. doi: 10.1186/1471-2105-12-402 (PMC3234255; doi:10.1186/1471-2105-12-402)
Supplement: Additional file 7 — Validation data set results comparison between MFP and Sanger sequencing. (A) Call rate improvement when comparing MFP analysis to GSEQ 4.1 for each of 5 validation samples. (B) Accuracy between total calls made by MFP analysis and Sanger sequencing for each of 5 validation samples. (C) Call type details for a total of 5 discrepant calls between MFP analysis and Sanger sequencing seen in 3 of the validation samples. Interestingly, 3 of the 5 discrepant calls involved the same SNP (A12307G) in three distinct samples (C5, C9, C11) that was detected by MFP. (D) Sanger-based electropherogram clearly shows the presence of the A > G variation, not at 12307, but at its neighboring position 12308, as shown here for validation sample C5. [file 1471-2105-12-402-S7.PPT]

## Slide 1
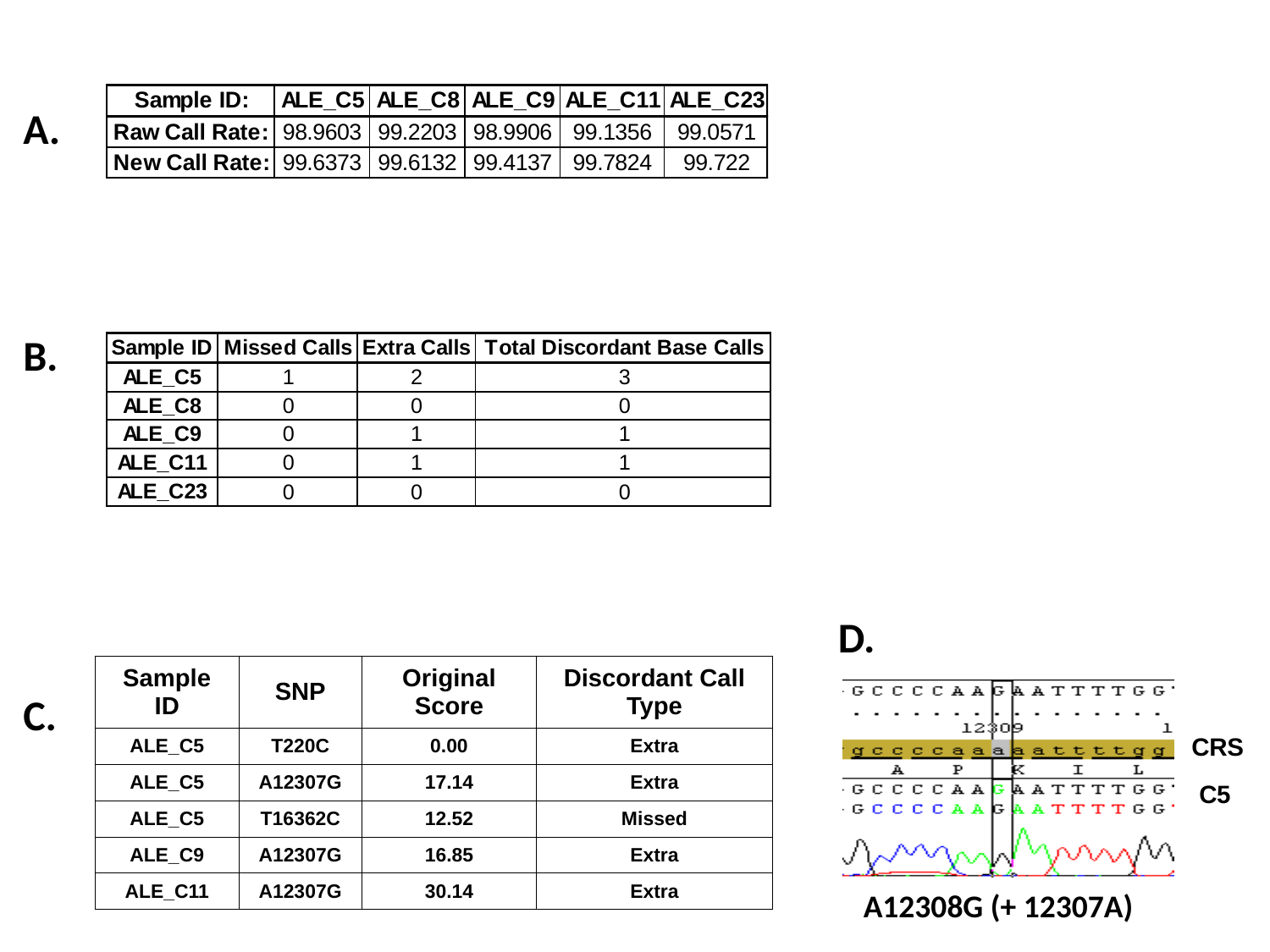

A.
B.
D.
| Sample ID | SNP | Original Score | Discordant Call Type |
| --- | --- | --- | --- |
| ALE\_C5 | T220C | 0.00 | Extra |
| ALE\_C5 | A12307G | 17.14 | Extra |
| ALE\_C5 | T16362C | 12.52 | Missed |
| ALE\_C9 | A12307G | 16.85 | Extra |
| ALE\_C11 | A12307G | 30.14 | Extra |
C.
A12308G (+ 12307A)
